# Is There a Role of Double Reporting and CT Pelvis for Lung Cancer Staging?

**DOI:** 10.4021/wjon492w

**Published:** 2012-04-23

**Authors:** Rajesh Botchu, Ganesh Retnasingam, Vimal Raj, Mohammad Ali Husainy, George Jakanani, Balaji Rao, James Entwisle

**Affiliations:** a Department of Radiology, GlenfIeId Hospitals, Glenfield, Leicester, UK

**Keywords:** Lung, Cancer, PET, Double, Reporting

## Abstract

**Background:**

Lung cancer is the most common cancer in the world. Staging of lung cancer involves CT of chest and abdomen. Subsequently these are discussed in MDT and if required PET imaging is arranged. We have performed a study to assess double reporting of the initial staging CT would identify in field metastasis and hence decrease the use of PET.

**Methods:**

A refined search from the lung cancer database over 2 years of 980 patients was performed. Metastasis identified on PET (SUV > 2.5) was nominated as the gold standard, 219 patients had both PET and staging CT (chest and abdomen) with 38 patients having metastasis on both PET and CT. CT images were reviewed by two independent radiologist who were blinded to the report. Identified metastases were graded if identified. These were grade as 1- definite, 2- equivocal, 3- normal. Subsequently through a process of arbitration a combined decision about the in field metastasis was achieved.

**Results:**

There were 21 metastasis which were within the field of chest and abdomen (in field metastasis). Only a half of these were identified by blinded observers. Following an arbitration there was no significant improvement in the pick up rate. There were 19 out of field metastasis in 15 patients out of this cohort. Majority of these (72%) were in the bony pelvis which would have been reported if a CT pelvis was performed as a part of staging. We estimate that one would have to perform 10 CT pelvises to save one PET-CT.

**Conclusion:**

Double reading of staging scan would not identify all infield metastasis. The increased contrast in PET images makes it easy to spot metastases. Hence there is no role for double reporting of staging CT in lung cancer management. Inclusion of pelvis in staging of lung cancer may be effective and would improve the detection of out of field metastases hence decreasing the use of PET.

## Introduction

Lung cancer is the most common fatal cancer in the industrialised world [1].Most pulmonary carcinomas are diagnosed at an advanced stage with less than 15% survival rate after 5 years [2].CT and PET scan are utilised to detect and decide treatment strategies of lung cancers. The literature has examined the economic cost of lung cancer in the United Kingdom after diagnosis [3]. We propose that inclusion of pelvis in staging CT scan and double reporting helps to identify most of the metastasis in lung cancer.

## Materials and Methods

From 2007 to 2009, all patients diagnosed with metastatic lung cancer who had both CT and PET were identified from the University Hospitals of Leicester Lung Cancer MDT database. Patients who did not have metastases or only underwent a single cross-sectional imaging modality (either PET or CT only) were excluded.

All patients underwent CT of chest and abdomen (Toshiba Aquilion 64) after intravenous contrast (Omnipaque 350mL). Chest was scanned with a 25 sec delay and abdomen was scanned till the iliac crest with a delay of 70 secs.

The site(s) of dissemination noted on initial staging CT and PET were documented separately. We classified metastasis into infield and outfield metastasis. The metastasis which could be identified on field of a CT of chest and abdomen were defined as infield metastasis and those which were outside this field were outfield metastasis. These were further classified depending upon the organ involved into liver, adrenal, spleen, lung, bone and others. Initial staging CT of patients were reviewed on AGFA, IMPAX 5.1 Morstel, Belgium work station retrospectively by two FRCR certified radiologists who were blinded to the report and subsequent studies. They were asked to identify the metastasis and grade them into 3 categories, 1 being 100% probability of metastasis, 2 being 50% probability and 3 being no metastasis. These were analysed for interobservor correlation and correlation with in field metastasis identified on PET. Metastasis noted on PET was used as the reference standard.

## Results

Total of 980 patients with lung cancer went through MDT at our university hospital in two year period 2007 - 2009, 219 patients underwent both PET and staging CT scan. A filtered search of the database revealed 38 patients with metastatic disease who underwent CT and PET scanning.

There was male predominance of 1.7:1 ( 24 men and 14 women). The mean age of the group was 70 yrs (range 55 to 83 yrs). Correlation was noted between PET and CT in 16 metastasis, 21 new metastasis were identified on PET which could be correlated retrospectively with initial staging CT. “In field” metastasis was defined as within the site covered by standard CT protocol. 19 metastasis were outside the field of coverage of staging CT chest and abdomen, herewith referred to as “out of field” metastasis, 15 of the out field metastasis were bony involving the pelvis, sacrum, Ilium, acetabulum and proximal femora (Table 1).

**Table 1:** Infield and Outfield Metastasis

Infield	21	Outfield	19
Liver	3	Ilium	5
Adrenal	5	Pelvis	4
Ribs	4	Acetabulum	2
Scapula	1	L4	1
Spine	1	High cervical nodes	1
Lung	2	Sacrum	1
Sternum	1	Proximal femur	2
Soft tissue	2	Glutei	2
Spleen	1	Post thigh	1
Subdiaphragmatic node	2		

Independent retrospective review by Fellowship trained radiologist identified half of the in field metastasis. Each radiologist identified10 and 11 lesions respectively out of the 21 in field metastasis.

## Discussion

The most common malignancy in the western world is bronchogenic carcinoma. The normal pathway of diagnosis of lung cancer includes, suspicion on chest radiograph and staging CT chest and abdomen. These are subsequently discussed in multidisciplinary meeting and patients are referred for PET.

There are various guidelines available to justify the use of PET scan clinically [4]. Recently, the joint project between the European Association of Nuclear Medicine (EANM) Oncology Committee and European Association of Nuclear Medicine (EANM) Physics Committee published its recommendations to provide the minimum standards for the acquisition and interpretation of PET and PET/CT scan with fluorine 18 fluorodeoxyglucose (FDG) [5].

In terms of specificity and sensitivity, PET scan results are not uniform across the spectrum of histological types of lung cancers. PET is recognised as an imaging modality that can quantify the aggressiveness of small lung adenocarcinomas by correlating the glucose metabolic rate of the neoplasm with the degree of (18F) - FDG uptake [6, 7]. However, the sensitivity of PET in detecting the disease burden of BAC remains controversial because of scant FDG uptake [8, 9].

PET is accepted as an important imaging tool to detect, stage, re-stage and access treatment response in cancers [10, 11]. New high resolution CT scanners enable the visualisation of morphological and anatomical structures with a greater degree of clarity. CT can be utilised to precisely localise lesions detected by PET.

Double reading has been used in breast cancer imaging with several studies reporting an increase in cancer detection by up to 15%. It also enables to increase the sensitivity [12-14]. Goddard et al had reported significant change (60%) in patient management following double reading of MR [15]. Murphy and colleagues had shown reduction in error by double reporting of CT colons [16].

**Figure 1:**
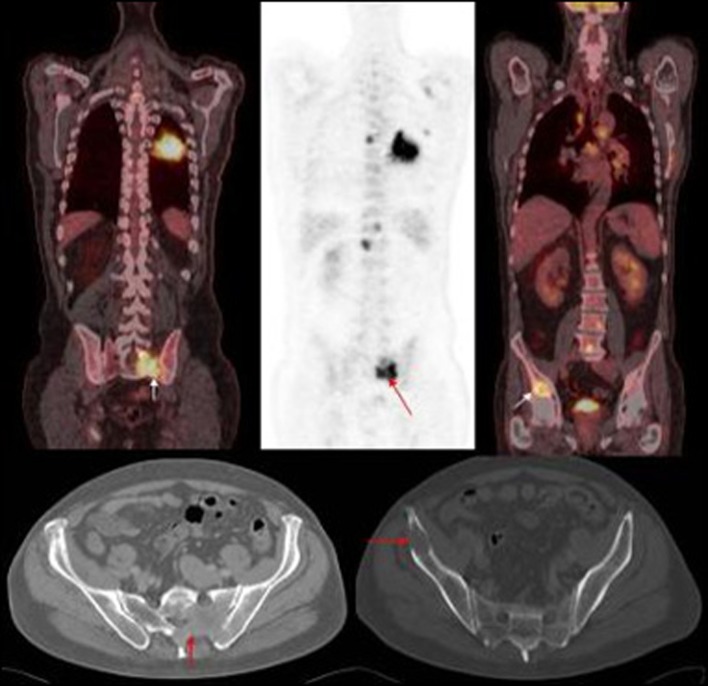
PET images of primary left lung cancer metastatic deposits in the bony pelvis and lumbar vertebrae with corresponding axial CT images of the pelvis.

**Figure 2:**
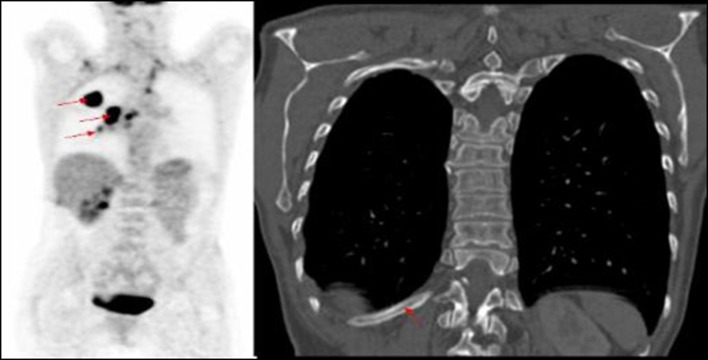
PET image of right lung tumour with right hilar mass and increased uptake in mid thoracic vertebra, not identified on corresponding CT of primary lung cancer.

**Figure 3:**
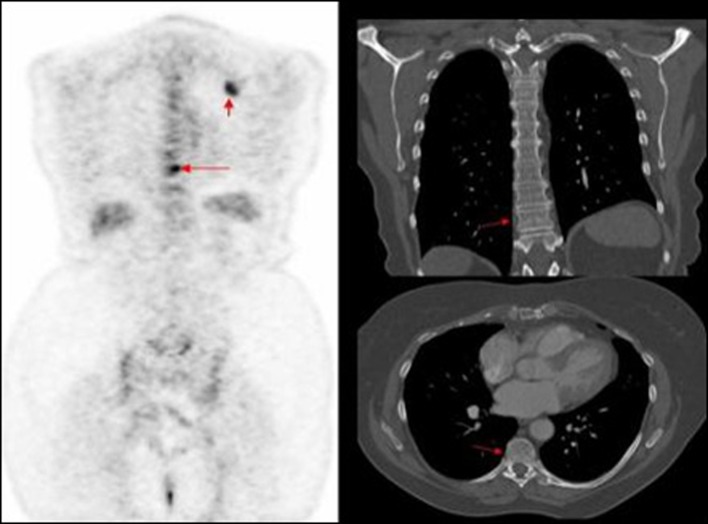
PET image of thoracic vertebrae and left scapula metastatic deposits, not identified on corresponding CT.

**Figure 4:**
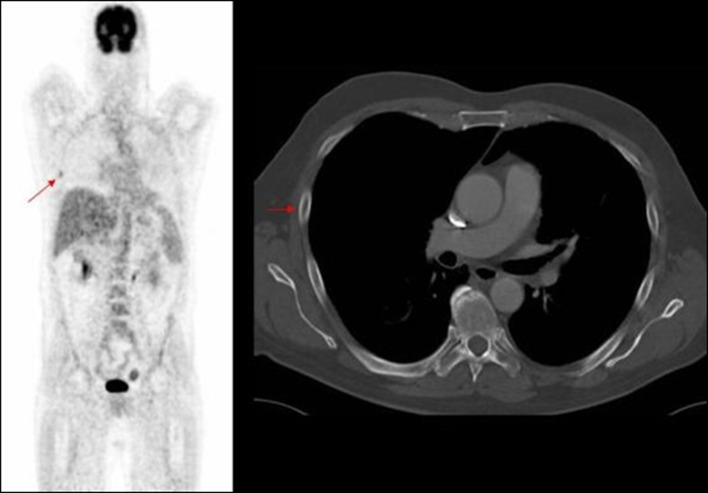
PET image of rib deposit initially noted in PET, not identified on corresponding axial CT image.

In our study nearly half of the in field metastasis were identified by two observers (Fig. 1). Most of the missed in field metastasis were bony involving the ribs, acromion and spine (Fig. 2, 3, 4). This could be explained by a number of reasons. Firstly it is easier to overlook a small bony metastasis especially in the ribs. Secondly, MIP (maximum intensity projection) reconstructions helps to depict the metastatic deposits better. If one does not routinely perform these, it might account for the missed deposits.

Out field metastasis were predominantly bony and hence can easily be demonstrated on CT of pelvis. Hence involving the pelvis as a part of routine staging scan in lung cancer would enable identification of occult skeletal deposits. The additional diagnostic benefit of extending the field of CT for the purpose of staging lung cancer has to be balanced against the additional individual radiation dose accumulation and the economic cost. However, our case series has demonstrated that the true extent of disease burden will not have been revealed with limited imaging. Hence the possibility of making a “wrong” decision is always present as long as uncertainty remains [17, 18]. The implications are far reaching in terms of inappropriate therapy influenced by diagnostic imaging and indirectly, cost of hospitalisation. This has significant impact on the ultimate care that the patient receives.

In conclusion, staging CT scan for lung cancer should include chest, abdomen and pelvis for adequate quantifiable pick up of metastasis. However, our study demonstrated that double reading would not pick up all in field metastasis. This is primarily due to increased sensitivity of PET over CT for assessment of smaller lesions.
